# Directed invasion of cancer cell spheroids inside 3D collagen matrices oriented by microfluidic flow in experiment and simulation

**DOI:** 10.1371/journal.pone.0264571

**Published:** 2022-03-01

**Authors:** Florian Geiger, Lukas G. Schnitzler, Manuel S. Brugger, Christoph Westerhausen, Hanna Engelke

**Affiliations:** 1 Department of Chemistry, Ludwig-Maximilians-Universität München, Munich, Germany; 2 Experimental Physics I, Institute of Physics, University of Augsburg, Augsburg, Germany; 3 Stiftung der Deutschen Wirtschaft (sdw) gGmbH, Berlin, Germany; 4 Physiology, Institute of Theoretical Medicine, University of Augsburg, Augsburg, Germany; 5 Center for NanoScience (CeNS), Munich, Germany; 6 Institute of Pharmaceutical Sciences, Department of Pharmaceutical Chemistry, University of Graz, Graz, Austria; Bioinformatics Institute, SINGAPORE

## Abstract

Invasion is strongly influenced by the mechanical properties of the extracellular matrix. Here, we use microfluidics to align fibers of a collagen matrix and study the influence of fiber orientation on invasion from a cancer cell spheroid. The microfluidic setup allows for highly oriented collagen fibers of tangential and radial orientation with respect to the spheroid, which can be described by finite element simulations. In invasion experiments, we observe a strong bias of invasion towards radial as compared to tangential fiber orientation. Simulations of the invasive behavior with a Brownian diffusion model suggest complete blockage of migration perpendicularly to fibers allowing for migration exclusively along fibers. This slows invasion toward areas with tangentially oriented fibers down, but does not prevent it.

## Introduction

Invasion from tumor spheroids is influenced by cellular signaling as well as by the properties of the extracellular matrix. The onset of invasion has been described as fluidization of the spheroid, which is triggered by a phase transition from a jammed state to an unjammed, fluid-like state [1]. After this transition and the accompanying onset of invasion, single cells often start to stream out of the collective. This has been described as a transition from fluid to gas-like state. Recent work has identified molecular mechanisms of the unjamming transition. One such mechanism is based on Rab5a activating the ERK1/2 pathway [2]. Other molecular influences are e.g. e-cadherins and in general an epithelial to mesenchymal transition [3]. Next to these cellular properties, characteristics of the extracellular matrix that drive such unjamming transitions have been identified. For example matrix density and extracellular confinement play a critical role in this transition [3].

Single cell migration is also strongly influenced by matrix properties, such as matrix density, mesh size, as well as fiber length and thickness [4–9]. Additionally, fiber alignment and orientation has been shown to impact single cell migration based on contact guidance and the non-linear rheological properties of the extracellular matrix [10, 11]. For invasion from a cell collective, such an influence of fiber alignment has been predicted from theoretical modeling [12]. Experimentally, short-range alignment has been shown to direct protrusions [13]. Furthermore, radially oriented fibers resulting from matrix remodeling of spheroids, lead to increased invasion [14, 15] and cells streaming along the oriented fibers [16]. Cell contractions however, only result in radial alignment and do not allow for a comparison of tangential and radial alignment. Thus, these experiments suggest that fiber alignment may also be an important factor that influences not only single cell migration, but also invasion from a cell collective as predicted from theory. Yet, detailed experimental investigation and description is still lacking mainly due to the challenge of achieving significant alignment including tangential and radial fiber orientation around cell spheroids.

Here, we investigate this influence of fiber alignment and orientation of tangentially versus radially oriented fibers on spheroid invasion. We use shear flow in a microfluidic channel during collagen polymerization to align collagen fibers of a matrix containing cancer cell spheroids. After polymerization of the gel, application of the external flow is stopped. The resulting gel structure shows highly aligned collagen fibers around the spheroids with radial and tangential orientation. Over time, the spheroids spread into the collagen gel. The observed invasion shows clear directionality along radially aligned fibers. Combining live cell imaging with particle image velocimetry measurements and finite element simulations, the fiber alignment resulting from shear flow and the subsequent shape evolution of cell spheroids in the aligned fibers can be described by a strongly enhanced invasion speed of cells moving along fibers compared to perpendicular to fibers.

## Results and discussion

### Alignment of collagen gels

**Fig 1:**
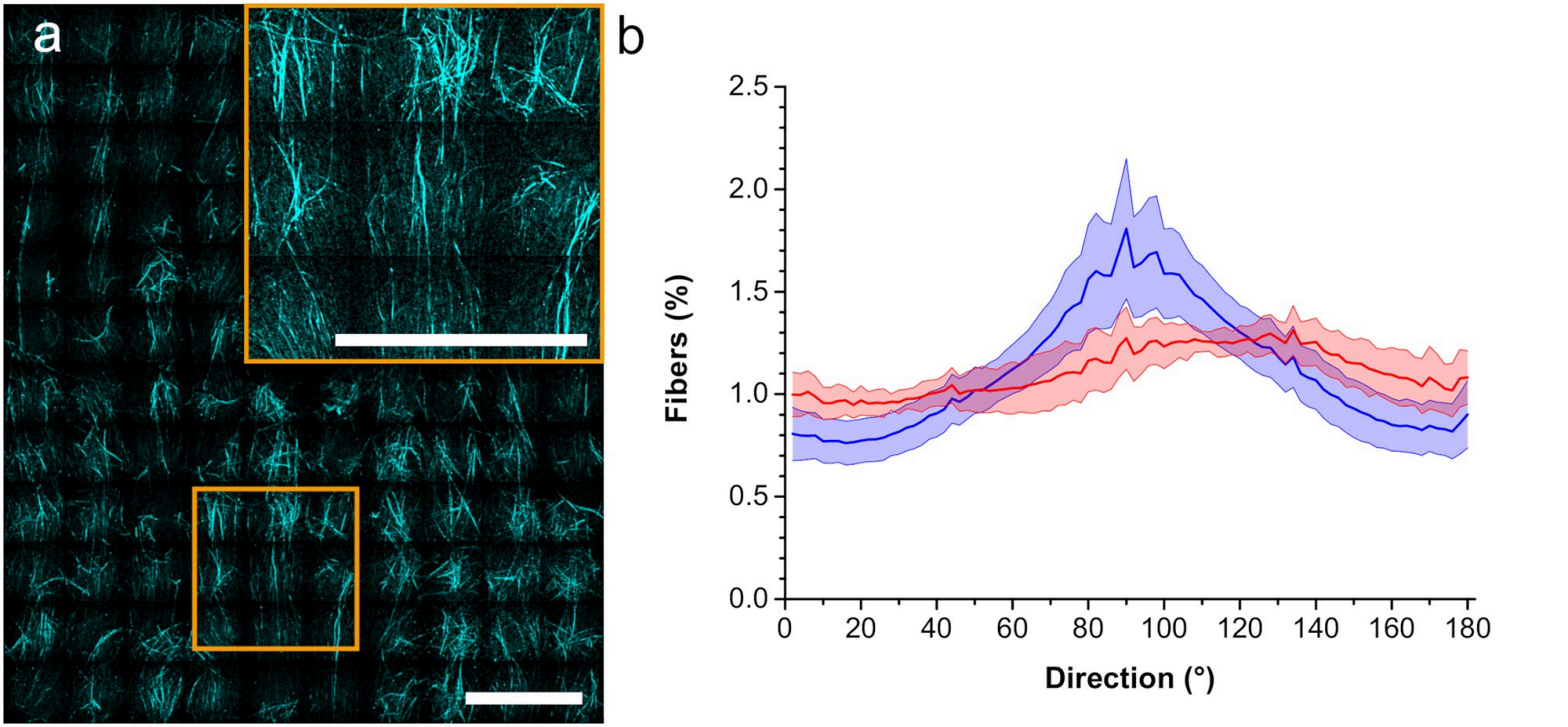
Alignment of collagen fibers. a) Collagen fibers aligned via microfluidic flow in a channel with 400 μm height. Insert: magnification of image marked with orange box. b) Orientation distribution of fibers in 33 gels aligned with microfluidics (mean values blue; standard error light blue) and in non-aligned gels prepared in a well (mean values red; standard error light red). Scale bars = 200 μm.

Shear flow in microfluidic channels can align collagen depending on flow rate and channel size [17]. Here, we used 400 μm high channels to accommodate spheroids embedded in collagen gels (S1 Fig). We applied a pulse of high flow rate to fill the channel with collagen including HeLa cell spheroids followed by a phase of low flow rate during polymerization on ice. After polymerization, we stopped application of the external flow. This process resulted in gels with rather long collagen fibers oriented along the direction of the applied flow as displayed in Fig 1a. Quantitative analysis of the distribution of fiber orientations (Fig 1b, blue data) revealed a clear maximum at 90 degrees, which corresponds to alignment along the main flow direction. A control experiment without flow and outside the channel to minimize shear flow shows a fairly uniform distribution of fiber orientations (S2 Fig and Fig 1b, red data).

### Collagen fibers around spheroid represent flow field

**Fig 2:**
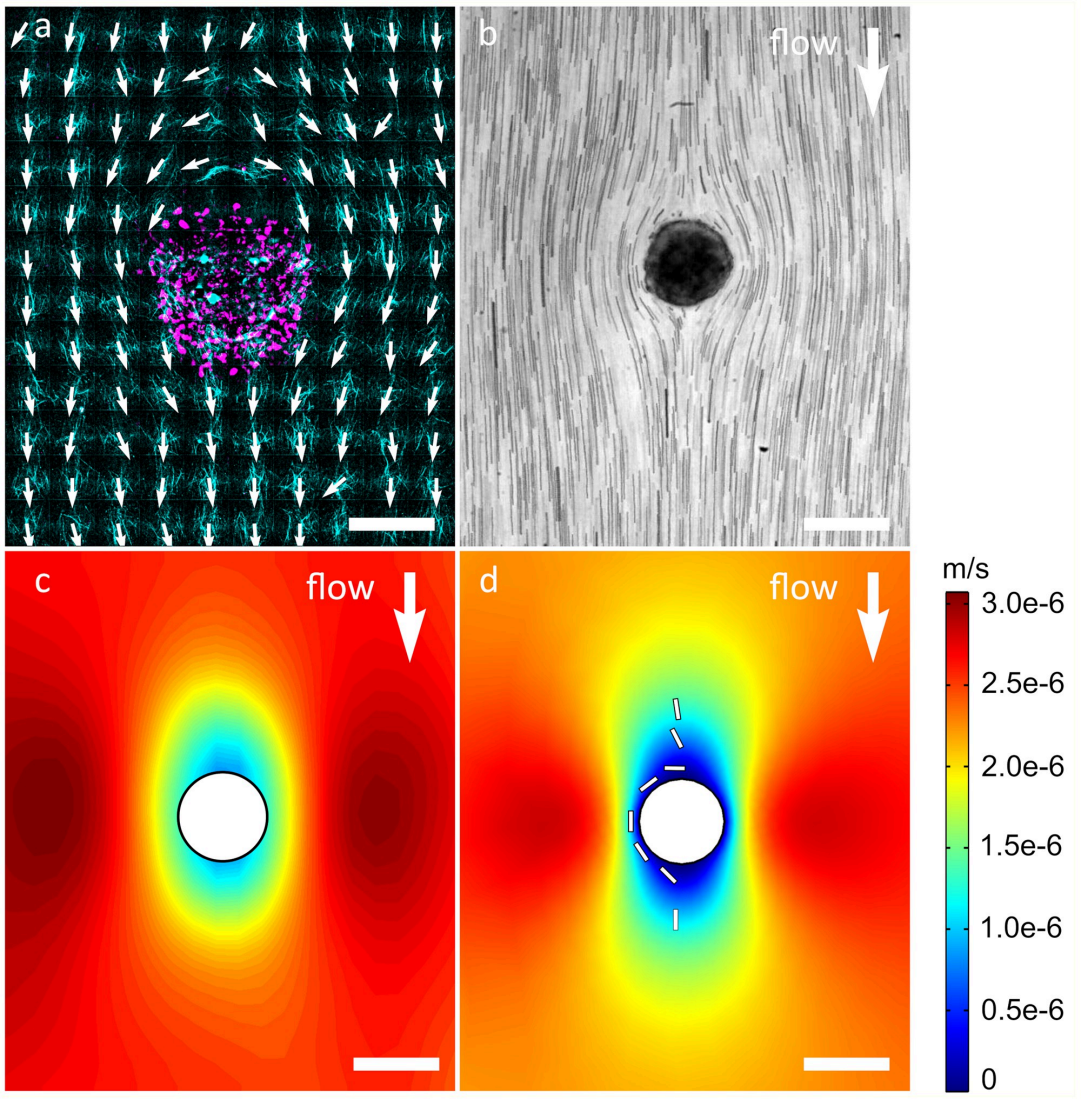
Characterization of shear flow and fiber alignment around the spheroid, while the main flow direction is from top to bottom. a) Spheroid (magenta) and aligned collagen fibers (cyan) with vectors in alignment direction (white) around the spheroid after polymerization. b) Flow trajectory of beads around a spheroid embedded in collagen during polymerization. The trajectory is the minimum projection of a time series of brightfield images. c) Velocity magnitude (color code) calculated from a particle image velocimetry measurement. d) Simulated velocity field (color code represents velocity magnitude) of the collagen mixture and the trajectory of a single collagen fiber (white bar) around the cell aggregate in the microfluidic channel. Scale bars = 200 μm.

Focusing on collagen alignment in proximity to spheroids, we find fiber orientations around the spheroid mostly along the stream lines that one would expect to result from the applied flow field around the spheroid. However, the collagen fibers at the spheroid side facing upstream with respect to the applied flow were oriented perpendicularly to the flow direction (Fig 2a). With respect to the spheroid, this lead to mostly tangential fibers around the spheroid with the exception of radial orientation at the side facing downstream of the main flow. To further characterize the flow field around the spheroid applied during polymerization and to understand the resulting fiber orientation after polymerization, we measured the flow in the region of the spheroid during collagen polymerization. Tracking of beads moving in the collagen gel during polymerization revealed traces as displayed in Fig 2b. Analysis of the velocity field with particle image velocimetry (PIV) provides a qualitative description of the force field applied by the shear flow. Fig 2c shows the direction and magnitude of the resulting velocity fields. It reveals a decrease in forces at the periphery of the spheroid–specifically at its sides facing up- and downstream of the main flow direction. The obtained velocity fields correspond very well to simulations of shear flow in a microfluidic channel around an immobilized sphere (Fig 2d and S3 Fig). Based on this confirmation that the flow simulation describes the conditions of the experiment, we next ran a simulation of a single fiber in the respective flow field around a spheroid. Fig 2d shows the orientation of the fiber in the simulation. While it is oriented along flow direction far away from the spheroid, the force distribution close to the upstream side of the spheroid orients the fiber perpendicularly to main flow and tangentially to the spheroid. It then continues to exhibit a tangential orientation with respect to the spheroid until it reaches the downstream side of the spheroid, where it is oriented radially with respect to the spheroid and along main flow direction. This corresponds very well to the fiber orientation of the gel measured around the spheroid (Fig 2a), suggesting that the forces of the flow are responsible for the obtained orientation of collagen fibers. In turn, the results also confirm that the collagen orientation displays and preserves the force field applied during polymerization.

### Invasion of the spheroid

**Fig 3:**
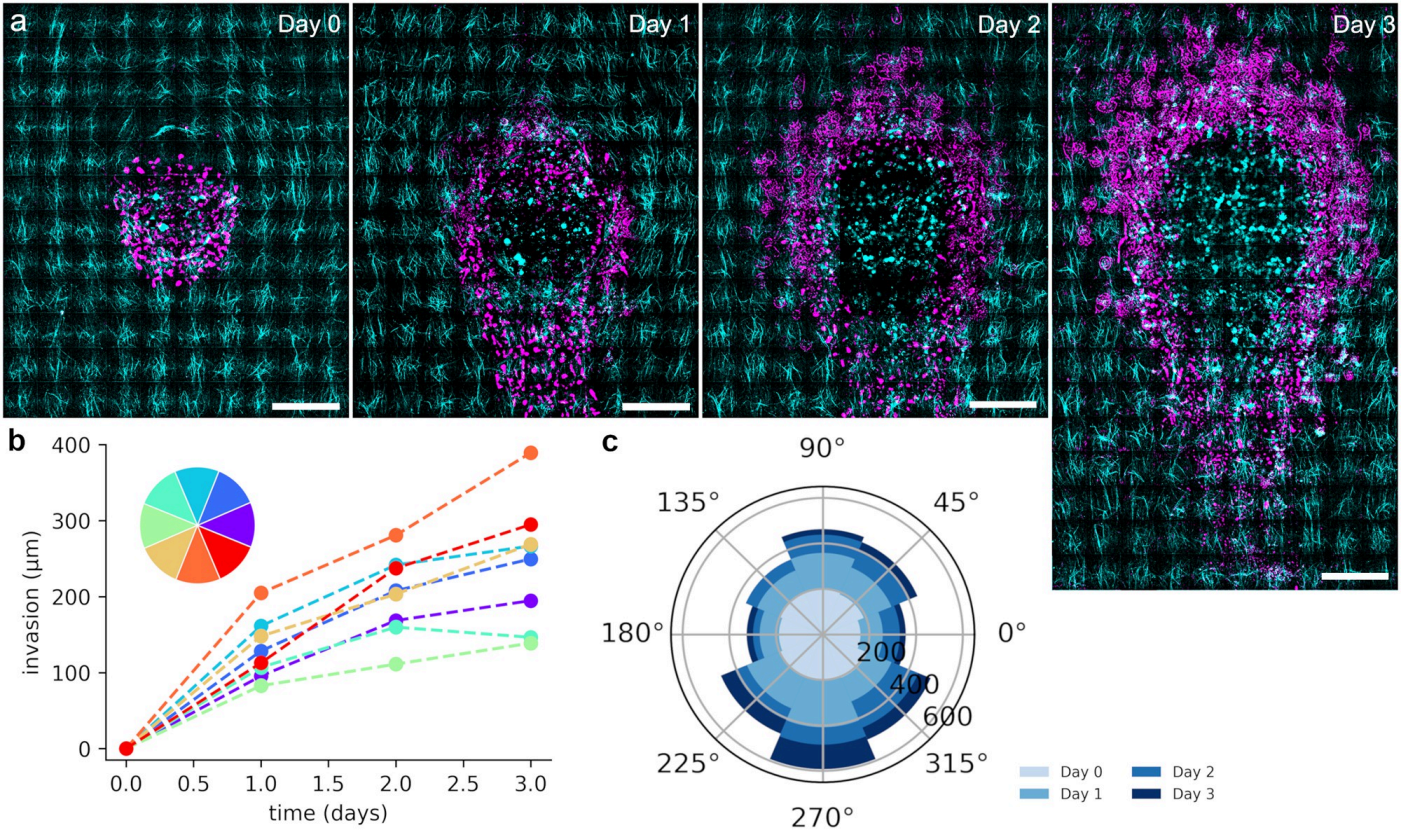
Invasion of spheroids. a) Microscopy images of spheroids invading into the surrounding aligned matrix over the course of 3 days. Note: the spheroid remains filled with cells, the void in the center of the spheroid is a result of image editing for better contrast in the composite with the matrix. For original images see S4 Fig. b) Mean relative invasion distance of 10 outmost cells in each direction (color-coded directions). The markers are averages of 20 aggregates. For error bars see supplementary information. c) Mean invasion distance of 10 outmost cells for different directions. The distance travelled per day in each direction is color coded in blue. Scale bars = 200 μm.

Next, we investigated the behavior of spheroids in the oriented hydrogels over time. The overall collagen concentration used is 1.85 mg/ml and allows for spontaneous invasion. Fig 3a shows that spheroids start to invade into the surrounding already one day after embedding them into the gels (see also S4–S6 Figs, S1 and S2 Movies). The invasion is much more pronounced along the radial fibers on the downstream side of the spheroid compared to the sides facing tangential collagen fibers. Over the course of 3 days, the observed invasion increases in all directions. Furthermore, we start to observe single cells streaming into the gel detached from the spheroid after two days. Independent of the direction, the rate of invasion is highest on the first day and slows down over time (Fig 3b). Quantitative analysis of the invasion into different directions (Fig 3b and 3c, S7 Fig) shows that cells invade furthest on the downstream side followed by the directions adjacent to downstream and the upstream facing direction. Least invasion occurs perpendicular to flow direction and thus on the sides of the spheroid facing tangentially oriented fibers. Here, the distance of invasion is about a factor of three less than on the downstream facing side with its perpendicularly oriented fibers. Collagen density is evenly distributed around the spheroid (S8 Fig). Thus, the directionality of invasion is a result of fiber alignment and the results suggest that invasion is promoted along fibers perpendicularly oriented with respect to the spheroid as compared to the direction across fibers tangentially oriented with respect to the spheroid. The spheroid fronts invading along main alignment direction face tangentially oriented fibers on the upstream side and radially oriented fibers on the downstream side. Therefore, we next analyzed upstream and downstream front of 10 spheroids into more detail.

### Influence of tangentially and radially oriented fibers on migration

**Fig 4:**
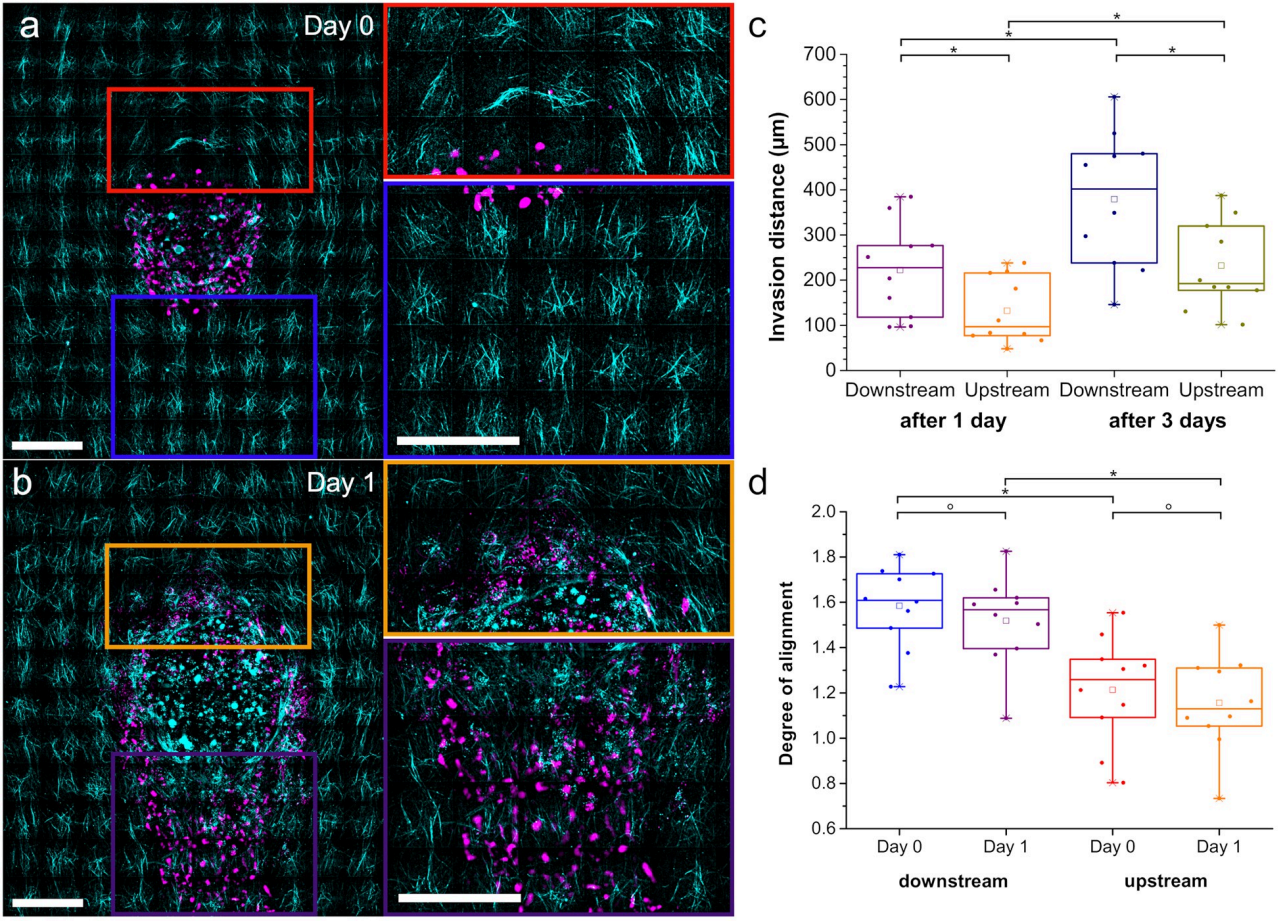
Invasion and fiber alignment on upstream and downstream front. a) Collagen fibers (cyan) and spheroid (magenta) at the upstream (red) and downstream (blue) side of the spheroid at day 0. b) Collagen fibers (cyan) and cells (magenta) at the upstream (orange) and downstream (purple) side of the spheroid at day 1. c) Position of invasion front with respect to initial spheroid surface (day 0) after 1 day and after 3 days on downstream and upstream side of 10 aggregates (*p < 0.05). d) Degree of alignment of the collagen fibers on downstream and upstream side of 10 aggregates at day 0 and day 1 (°p > 0.05, *p < 0.005). Scale bars = 200 μm.

Fig 3 clearly shows increased invasion on the downstream side of the spheroid compared to the upstream facing side. Focusing on these two sides of the spheroid (Fig 4a and 4b) we find that the distance the invasion front of the 10 spheroids analyzed into detail travels on average during the first day is much longer (222 ± 33 μm) for the downstream front compared to upstream (132 ± 23 μm) (Fig 4c). Also after three days, the downstream front shows similarly increased invasion compared to the upstream front (2.3 times increased distance of downstream compared to upstream after one day and 2.1 times after three days). Interestingly, the distance that the invasion front travelled after three days is in both directions only about a factor of 1.9 longer than after one day suggesting a non-linear time-evolution. Since the conditions of the two spheroid sides are the same with the only exception of the collagen fiber orientation with respect to the spheroid, we also analyzed the degree of alignment at the invasion fronts (see marked areas in Fig 4a and 4b, S9 Fig). The degree of alignment is calculated as the amount of fibers aligned in main flow direction in the analyzed gel relative to that of a gel without alignment exhibiting uniform (i.e. random) fiber distribution (see Materials and methods). Hence, a high amount of fibers oriented in flow direction will have a high degree of alignment, while areas with fibers oriented mainly perpendicular to flow direction will have a low degree of alignment. As expected from the microscopy images, the degree of alignment in main flow direction is much higher on the downstream side with the radially oriented fibers as compared to the upstream side with the tangential fibers (Fig 4d). The degree of alignment does not change significantly between day 0 and day 1. Comparing the results on invasion of upstream and downstream front of the spheroid confirms that a high degree of alignment of collagen fibers in radial direction promotes invasion compared to tangentially oriented fibers.

### Simulation of invasion behavior

**Fig 5:**
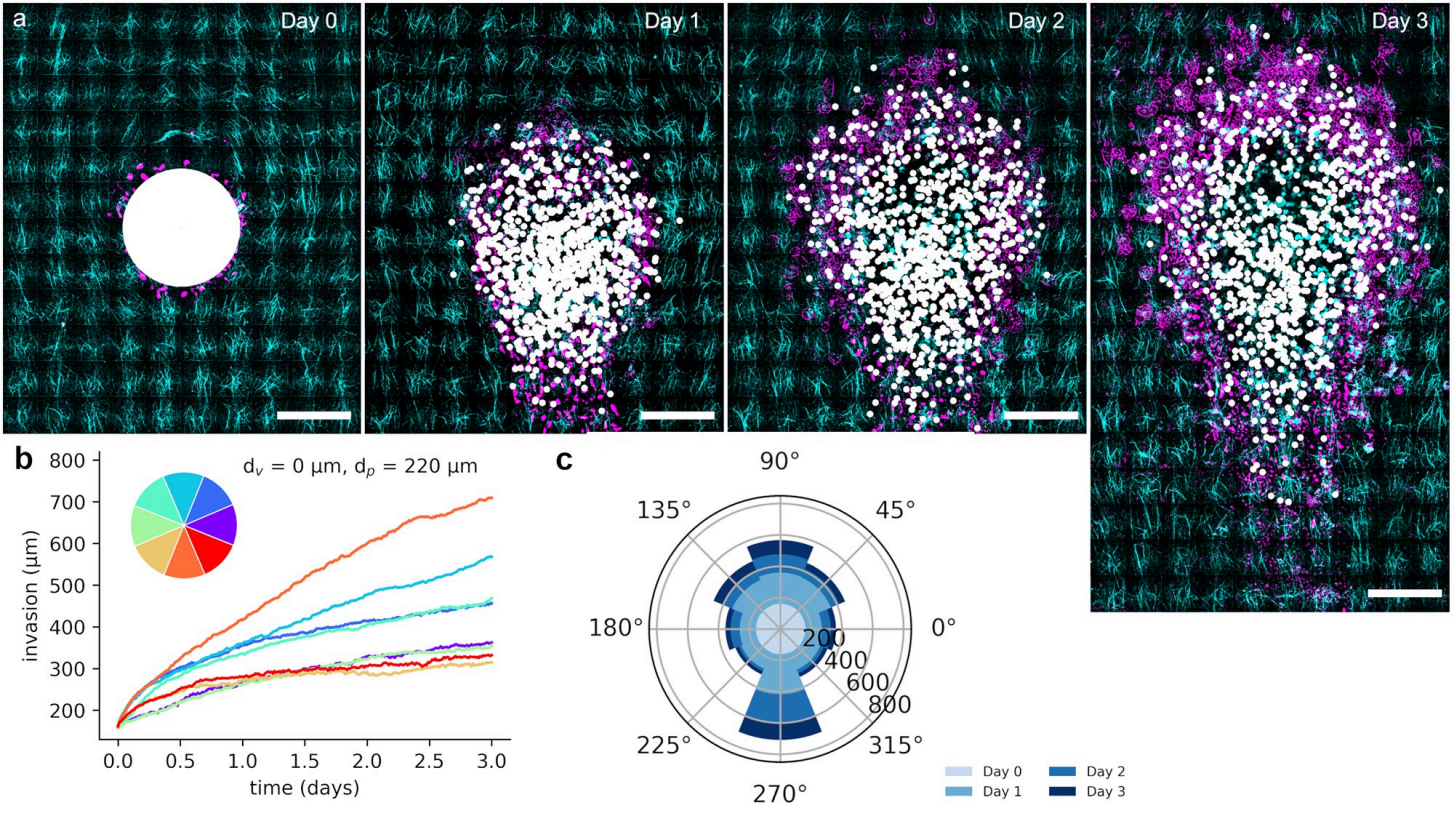
Simulation of invasion. a) Overlayed images of cell invasion in the collagen gel (cyan) during the experiment (magenta) and the simulated migration of cells (white) in a similar environment over the course of 3 days. b) Invasion distance of 10 outmost cells in each direction (color-coded directions) obtained in the simulations. For error bars see supplementary information. c) Invasion distance of 10 outmost cells in different directions obtained in the simulations. The distance travelled per day in each direction is color coded in blue. Scale bars = 200 μm.

Having established that the aligned collagen fibers and specifically their orientation strongly influence invasion of the spheroids, we next asked whether the observed invasion process can be described by a model, in which we assume that cells can invade along fibers faster than compared to invasion perpendicular to fibers (Fig 5). We assume a very simple model of cells distributed on a sphere the size of a spheroid (Fig 5a, Day 0). They are performing a random walk with some step size parallel and a smaller step size perpendicular to the fibers as derived from the traveled distance of the downstream and upstream front, respectively, on the first day. Fiber direction is determined and interpolated from the experimental data displayed in Fig 2a. We reduce the model to include the influence of fiber orientation on cell migration and neglect cell overlap, cell-cell contacts and resulting jamming effects. Furthermore, we do not take proliferation into account, since it is expected to be very slow in spheroids in 3D-environments leading to an estimated increase in radius of about 1.2 over the entire 3 days. Nevertheless, potential effects of proliferation on the invasion behavior cannot be excluded, specifically, such that result from an increased proliferation due to influences by the matrix. The shape resulting from the simulation with the initial step size parameters did not match the shape evolution measured in the experiment very well (S10 Fig). The simulation data show very little asymmetry in shape and the difference between up- and downstream invasion is much smaller than in the experiment. To increase asymmetry and thus the difference between up- and downstream invasion, we next reduced the step size perpendicular to fiber orientation, while maintaining the step size derived from the experiment for movement parallel to fibers. Expectedly, the decrease in perpendicular step size increased the asymmetry in shape and the difference in travel distance of the invasion fronts up- and downstream (S10 Fig). Strikingly, the best match in spheroid shape between experiment and simulation was obtained with a perpendicular step size of 0, i.e. complete blockage of migration perpendicularly to fibers (Fig 5, S10 Fig). This simulation assuming complete blockage of perpendicular migration describes the experimentally obtained shapes remarkably well–including invasion towards areas with fibers tangentially oriented toward the spheroid. Also the differences in travel distance of upstream and downstream front as displayed in Fig 4 are recapitulated with this parameter set by the model. Since the step size was calculated from the values of Fig 4 for the distance travelled during the first day for the movement along fibers, the numbers fit very well at that time. The distances after three days are slightly larger in the model compared to the experiment. This might be due to an overestimation of the step size based on effects of the collective, such as jamming, which decrease with increasing amount of single cells at the front moving detached from the collective, leading to a decreased front speed. However, the differences are not large given the error bars. Overall, the model suggests an increase in travelled distance with the square root of time, which results from the diffusive character. The values measured in the experiments (Figs 3 and 4) are in accordance with this increase. Comparing the angle distribution of invasion in experiment and simulation (Figs 3c and 5c, S5 Fig), the distributions match fairly well with the exception of the directions adjacent to the downstream direction (225° and 315°). This deviation may originate in the assumptions of the model, which neglects finite cell volume, proliferation, cell-cell interactions, remodeling of the matrix, and allows for overlap of cells. Significant remodeling of the matrix could not be observed and proliferation should not lead to such strong asymmetry, thus most likely these factors play a minor role in generating the observed deviation of simulation and experiment. In fact, the finite cell volume and cell-cell-interactions as well as exclusion of overlap are most likely main contributors to this deviation. Crowding effects at the bottle-neck that cells need to pass in order to reach the region of migration-promoting fibers, might lead to the experimentally observed enhanced migration in the sections next to the downstream direction compared to the simulation results.

## Conclusion

The fiber alignment with microfluidic flow during collagen polymerization that we describe here, allows for significant alignment in main flow direction. Analysis of the flow profile shows that the polymerized collagen gel preserves the flow field such that the fiber orientation mirrors the streamlines of the flow with the exception of the area around the upstream facing side of spheroids embedded in the collagen gel. The entire fiber orientation–including the upstream side of spheroids—can be described by the orientation of rods in the flow field of the applied flow. Thus, in principle, simulations of flow and of the effect of the resulting flow field on rod orientation should allow for prediction of fiber orientations depending on the applied flow field providing a predictable platform of fiber alignment.

The fiber orientation around spheroids resulting from the microfluidic alignment allowed us to study the influence of fiber orientation–particularly of tangential versus radial orientation with respect to the spheroid. This orientation remains fairly constant over time—we do not observe strong changes in the degree of fiber orientation, which have been observed previously mainly in 2D settings or between neighboring spheroids [16], although the invasion of the entire spheroid does lead to slight, local remodeling not affecting the average degree of orientation in our experiments.

Spheroids in the aligned collagen gels invaded readily into the gels. Such invasion processes have been described as transition from a jammed state to an unjammed, fluid-like state followed by isolation of single cells in a gas-like state. In immuno-fluorescence images we see characteristics of jammed cells inside the spheroid (S11 Fig, red box), while they show more elongated and less adherent structures at the periphery of the spheroid resembling an unjammed state. Starting on day 1, we also observe single, isolated cells around the spheroid.

The influence of fiber alignment on invasion is very pronounced and leads to a bias in invasion along fibers oriented radially with respect to the spheroid. In a first approximation, the resulting shape of the invading spheroid and its evolution over time was described by a model assuming Brownian diffusion with larger step size along fibers compared to that perpendicular to fibers. The resulting simulations describe the shape evolution of the spheroid obtained in the experiments remarkably well. Strikingly, parameter sets with no invasion perpendicular to the fiber orientation fit best with the experimental data. Thus, the origin of the invasion bias toward radially oriented fibers may indeed be a strong bias towards faster invasion along radially oriented fibers compared to tangentially oriented fibers. Further analysis and refinement of the model may show the influence of finite volume, jamming, slippage, proliferation and other effects, which may provide more insight into the influence of fiber orientation on invasion. This could also include modeling of the fiber orientation as a distribution with some finite width. Moreover, it may e.g. decipher whether the effect of fiber orientation is mainly due to contact guidance or influenced by collective effects as has been described for unidirectional invasion [18].

We observe some single cells, which are elongated along the fibers, however many invading single cells as well as the fronts of the collective do not show a clear cell orientation. Thus, the reason for the bias in invasion along radially oriented fibers may partially be based in polarization, but other factors most likely contribute to the effect, too. One such factor might for example be the enhanced force propagation resulting from contractions along radially oriented fibers compared to tangential fibers due to the non-linear stress-strain relation of collagen [19, 20]. As for the molecular mechanisms underlying the invasion, nuclear YAP (Yes-associated protein) has been shown to be able to trigger invasion [21, 22] and YAP’s translocation into the nucleus acts as mechanosensor [23]. In our case, however, it is mostly cytosolic and thus not active at the measured time of 3 days after embedding the spheroid in the gel (S11 Fig). Hence the underlying mechanisms of the biased invasion towards fibers oriented radially with respect to the spheroid remain to be investigated. The microfluidic platform we presented here, offers a well-defined system for such investigations. Understanding directional bias of invasion and its origins will not only be useful in cancer research, but also in many other fields such as tissue engineering, developmental biology and wound healing.

## Materials and methods

### Cell culture and spheroid formation

HeLa cells were cultivated in liquid Dulbecco’s modified Eagle’s medium (DMEM, Gibco), supplemented with 10% fetal bovine serum (FBS, Gibco) and 1% Penicillin Streptomycin (Gibco), at 37°C in a 5% CO_2_ atmosphere.

For HeLa cell spheroid formation, 500 cells were seeded in a 96-well plate with ultra-low adhesion (Corning) and incubated at 37°C and 5% CO_2_ for 48 h until the spheroid reached the desired size. At a diameter of 250–350 μm, the spheroids were transferred to a collagen gel mixture.

### 3D antibody staining

The primary and secondary antibodies used were YAP1 polyclonal rabbit antibody (PA1-46189; Thermo Fisher Scientific) and Donkey anti-Rabbit IgG (H+L) Highly Cross-Adsorbed Secondary Antibody, Alexa Fluor 546 (A10040; Thermo Fisher Scientific).

HeLa spheroids embedded into collagen were fixed with 4% PFA for 40 minutes and washed with PBS twice for 20 minutes. The cells were permeabilized for 20 minutes with 0.5% Triton X-100 in PBS and subsequently washed with PBS for 30 minutes. The cells were blocked with 1% BSA in PBS overnight. Primary antibodies were diluted 1:100 with 1% BSA in PBS and cells were incubated for 72 hours. Prior to incubation with secondary antibodies (1:200 in 1% BSA), the cells were washed twice with PBS for 30 minutes. The cells were incubated with secondary antibodies for 48 hours. Afterwards, the cells were washed with PBS for 30 minutes and Hoechst 33342 (0.5 μg/ml) for 40 minutes. Prior to imaging, the cells were washed again with PBS for 30 minutes. Finally, the PBS was renewed and kept in the reservoirs during confocal microscopy.

### 3D collagen matrices

Collagen gels were all prepared with the same compounds, which were kept on ice (except for the HeLa cells). Rat tail collagen I stock solution (Corning) was mixed with 1 μg ATTO-633 (NHS-Ester, ATTO-TEC) per 1.37 mg collagen to stain and later visualize the 3D network of the gels. Then, they were neutralized with Sodium hydroxide (1 N, Fluka) and diluted with Dulbecco’s phosphate-buffered saline (DPBS(1x), Gibco) until the desired collagen concentration (3.32 mg/ml; final: 1.85 mg/ml) was reached. Finally, three HeLa cell aggregates, diluted in DMEM-Medium (45% of the final volume), were added.

The gel mixture was immediately filled in a μ-Slide VI 0.4 ibiTreat (Ibidi), which was connected to a LA-120 syringe pump (Landgraf). The mixture was sucked with 90.2 μl/min into the channel of the slide, until an aggregate reached the channel. Then the draw speed of the syringe pump was reduced to 0.2 μl/min during polymerization on ice. After 30 minutes the slide was handled at room temperature and 0.1 μl/min pump draw speed. After 15 minutes the gel was transferred to 37°C, without the syringe pump, to finish the gelation process. After a successful gelation all gels were overlaid with DMEM, to prevent them from drying out.

### Analysis of the microfluidic chamber

The flow field in the channel was analyzed experimentally with digital particle velocimetry (DPIV) as well as numerically with finite element method (FEM) simulation. For the experimental characterization the same protocol as described in the part “3D collagen matrices” was used. In addition a latex microsphere suspension (Polybead Carboxylate Microspheres 3.00um, Polysciences Inc., Warrington, PA, USA) was added (1% of the final volume) to the HeLa cell aggregates. For the analysis, we used light microscopy in combination with a CCD camera (FASTCAM 1024PCI, Photron, Ottobrunn, Germany). The movement of the particles was recorded with a frame rate of 1 fps at a constant flow rate of 0.2 μl/min. A MATLAB (R2017b, The MathWorks Inc., Natick, MA, USA) script based on the open source PIVlab (version 2.37) toolkit was employed to extract the two-dimesional velocity profile [24, 25]. FEM simulation was done with the commercially available software COMSOL Multiphysics (5.6, Comsol Inc., Burlington, MA, USA). The Navier-Stokes equation together with the continuity equation is solved for a stationary 2D model of the channel in the laminar flow interface of the Computational Fluid Dynamics (CFD) module. The width and the length of the channel is 3.8 mm and 17 mm respectively. The height of the channel (0.4 mm) was taken into account by applying a shallow channel approximation. At the inlet of the channel a laminar inflow boundary condition with a flow rate of 0.2 μl/min and at the outlet a static pressure condition was applied. The size and location of the HeLa cell aggregate (diameter d = 208 μm) in the channel is taken from the experiment. A physics controlled mesh was used with element size ‘normal’. By coupling the CFD module together with the Solid Mechanics module via a Fluid Structure Interaction (FSI) we also simulated the time dependent trajectory of a fiber in the flow field. For the CFD module the same parameters were used as in the stationary study. The fiber is assumed to be rigid and has a length of 47.5 μm and a width of 5 μm. A physics controlled moving mesh was used with element size ‘normal’.

### Spinning disk confocal microscopy

Confocal microscopy for live-cell imaging was performed on a setup based on the Zeiss Cell Observer SD utilizing a Yokogawa spinning disk unit CSU-X1. The system was equipped with a 1.40 NA 63x Plan apochromat oil immersion objective from Zeiss. The setup was heated to 37 °C and a CO_2_ source was provided to keep the atmosphere at 5% CO_2_ during the measurements. The resulting images were processed with the Zen software by Zeiss. Cell spheroids were imaged in brightfield mode with a tungsten-halogen lamp with 682.0 μW and 200 ms exposure time. For fluorescence images, Hoechst 33342 was excited with a 405 nm laser at 11.2 μW intensity for 200 ms and Alexa Fluor 546 of the secondary YAP antibody with a 561 nm laser at 70.7 μW intensity for 1000 ms. Images with ATTO-633 dye were taken with a 639 nm laser at 5.5 μW intensity and 200 ms exposure time. Each image consisted of 120 to 160 pictures, depending on the spread of the spheroid over time, and has an 11 frame z-stack with a 1.5 μm distance between z-planes. In the excitation path a quad-edge dichroic beamsplitter (FF410 /504/582/669-Di01-25x36, Semrock) was used. Band-pass filters 525/50 and 690/60 (both Semrock) were used in the detection path. Separate images for each fluorescence channel were acquired using two separate electron multiplier charge coupled devices (EMCCD) cameras (PhotometricsEvolve^™^).

### Image analysis

Analysis of fiber orientation was performed with ImageJ [26, 27]. Migration distances and aggregate axes were analyzed with Imaris (v 8.2.0, Bitplane, AG Zurich, Switzerland) and further processed with Microsoft Excel (version 2010). Statistics and data presentation was done with OriginPro (Version 8.0891/9.0.0, OriginLab Corporation, Northampton, MA, USA). Normality was tested with a Shapiro-Wilk test and statistical significance with the non-parametric Kruskal-Wallis test. Boxplots show mean (square), the box consisting of median, lower and upper quartile (25th and 75th percentile), whiskers (5th and 95th percentile), and outliers (marked x). The degree of alignment was introduced to quantify the orientation of the collagen gel along the main flow direction. Orientation angles of all fibers were determined using a set of Gaussian blur and Rolling ball filters, thresholding, and the local gradient method of the imageJ directionality plugin. Fibers between 68° and 112° (90°± 22°) are considered aligned with the main flow, which has an orientation of 90°. The degree of alignment was calculated as the percentage of aligned fibers of the analyzed gel divided by the percentage of aligned fibers calculated for a gel with uniform fiber distribution (25.56%). Thus, it measures the ratio of fibers oriented in main flow direction in the analyzed gel relative to a non-oriented gel. The invasion distance was determined by the difference of the average position of the 10 cells, which travelled furthest into the collagen gel into the direction of interest at day 0 and day 1 or day 3 respectively. The brightfield images of cell aggregates presented together with collagen fibers were processed with ImageJ [26, 27], for better visibility of both channels in one picture. A set of gaussian blur (sigma = 1), subtract background and despeckle was applied three times, with a decreasing value for the rolling ball radius used in each cycle (rolling = 200, 100 and 50). Finally, the image was pseudo-colored in magenta by changing the LUT from gray values to magenta. Unprocessed pictures of all used aggregates can be found in the SI.

### Simulation of invasion

In order to get a better understanding of the invasion of HeLa cell aggregates after collagen gel polymerization we introduced a numerical model, which is based on a simple 2D random walk algorithm. For the simulations we used the Python programming language (Python 3.8.3, Python Software Foundation). In our model we make two assumptions. First, we assume that the invasion is a diffusion-like process, which we model by a random walk. Second we assume that the step size is location- and direction-dependent. This dependency is given by the fiber orientation, which can be extracted from the experiment. Therefore we divided the field of view into boxes (*d* = 103 μm) and calculate the mean fiber orientation for each box. At each time step there are four ways with the same probability how a single cell can move: Parallel or perpendicular to the fiber and for both options the two possible directions. A step parallel to the fiber orientation results in a shift of the location of:

$$\Delta x=\pm k_{p}\text{cos}\left(\alpha \right)\;\text{and}\;\Delta y=\pm k_{p}\text{sin}\left(\alpha \right),$$

Where *k*_*p*_ is the step size parallel to the fiber orientation and *α* is the fiber orientation at the location. The positive and negative sign indicates in which direction with respect to the fiber orientation the step is made. A step perpendicular to the fiber orientation results in a shift of the location of:

$$\Delta x=\pm k_{v}\text{cos}\left(\alpha +90{}^{\circ}\right)\;\text{and}\;\Delta y=\pm k_{v}\text{sin}\left(\alpha +90{}^{\circ}\right),$$

Where *k*_*v*_ ist he step size perpendicular to the fiber orientation. We estimate the step size *k*_*p*_ and *k*_*v*_ from the data of the experiment:

$$k_{p}=\frac{r_{p}}{\sqrt{N}}\;\text{and}\;k_{v}=\frac{r_{v}}{\sqrt{N}},$$

Where *r*_*p*_ is the distance the cells travelled within one day downstream where most of the fibers are oirentated parallel to the invasion, *r*_*v*_ is the distance the cells travelled within one day upstream where most of the fibers are oirentated perpendicular to the invasion and *N* is the number of time steps.

## Supporting information

S1 Fig Schematic drawing of the microfluidic channel. Dimensions as stated; drawing not to scale.
(TIF)

S2 Fig Non-aligned collagen fibers synthesized in a well without microfluidic flow. Scale bar = 200 μm.
(TIF)

S3 Fig Characterization of the whole microfluidic channel with 4x magnification. a) Brightfield image of a cell aggregate embedded in a collagen bead mixture without applied flow. b) Calculated velocity magnitude with flow vectors of the beads. c) Flow trajectory of beads around a cell aggregate embedded in collagen during polymerization. d) Simulated velocity field of the collagen mixture around the cell aggregate in the microfluidic channel. e) Zoom in on the immediate surrounding of the cell aggregate in the simulation. Scale bars = 500 μm.
(TIF)

S4 Fig Original brightfield (BF) images of the spheroid displayed in Figs 2–4. Scale bars = 200 μm.
(TIF)

S5 Fig Measured invasion with error bars. a) and b) Data from experiment: a) Invasion distance of 10 outmost cells in 4 main directions (color-coded directions). The markers are averages of 20 aggregates, errors are standard error of the mean. b) Invasion distance of 10 outmost cells for different directions. The distance travelled per day in each direction is color coded in blue. c) and d) Simulation data (step size parallel to fibers dp = 220 μm, perpendicular to fibers dv = 0 μm): c) Invasion distance of 10 outmost cells in 4 main directions (color-coded directions). The lines are averages of 10 simulations, errors (shades) are standard deviations. d) Invasion distance of 10 outmost cells for different directions. The distance travelled per day in each direction is color coded in blue.
(TIF)

S6 Fig Invasion of spheroids in aligned matrix. The displayed spheroids provide an overview of experimental variability. a) Spheroid showing a fairly low degree of asymmetry. b) Analysis of directionality of invasion of spheroid displayed in a). c) Spheroid showing fairly strong asymmetry of invasion. d) Analysis of directionality of invasion of spheroid displayed in c). Scale bars = 200 μm.
(TIF)

S7 Fig Boxplots of the invasion distance dependent on angle segment. Relative invasion distance travelled by outmost cells within first day, first 2 and 3 days. The respective mean values are represented in Fig 3b.
(TIF)

S8 Fig Density distribution of the collagen fibers around the spheroid. The intensity of the representative fiber image shown in Fig 3 on day 0 at various binnings to assess the density distribution of collagen. a)-c) show maximum intensity binning at various binning factors. d)-f) show mean intensity binning at various binning factors. These exemplary intensity distributions show that the fiber density is homogeneous around the spheroid and not affected by the flow.
(TIF)

S9 Fig Orientation distribution of the collagen fibers on up- and downstream front of the spheroid. Orientation distribution of collagen fibers in the areas (as marked in Fig 4) around the upstream front of the spheroid at day 0 (red) and day 1 (orange) and around the downstream front at day 0 (blue) and day 1 (purple).
(TIF)

S10 Fig Simulation sweep with step sizes perpendicular to fiber orientation (r_v) per day of 0, 60, 120 and 220 μm respectively. a) Overlayed images of cell invasion in the collagen gel (cyan) during the experiment (magenta) and the simulated migration of cells (white) in a similar environment over the course of 3 days. Parallel step size (r_p) = 220 μm b). Invasion distance of 10 outmost cells in 4 main directions (color-coded directions). d) Invasion distance of 10 outmost cells for different directions. The distance travelled per day in each direction is color coded in blue. Scale bars = 200 μm.
(TIF)

S11 Fig Immunostaining of invading spheroid. a) Spheroid fixed after 3 days in an aligned collagen gel in a microfluidic channel. The images show brightfield (BF), YAP immuno-staining (YAP, yellow), the nuclei stained with Hoechst 33342 (cell nuclei, blue), and a composite of YAP and Hoechst. b) Zoom-in of YAP stained in cells at the spheroid periphery (blue box) and in the center of the spheroid (red box). c) Ratio of YAP intensity in the cell nuclei and cytosol for cells at the periphery (blue) and cells at the aggregate center (red); p < 10–4.
(TIF)

S1 Movie Time-lapse brightfield images of a spheroid in an aligned collagen gel over the course of 70 h. (MP4)

S2 Movie Time-lapse images of a spheroid (magenta) and the surrounding aligned collagen gel (cyan) over the course of 70 h. (MP4)

## References

[1] Oswald L, Grosser S, Smith DM, Käs JA. Jamming transitions in cancer. J Phys D Appl Phys. 2017;50(48):483001. doi: 10.1088/1361-6463/aa8e83 29628530 PMC5884432

[2] Palamidessi A, Malinverno C, Frittoli E, Corallino S, Barbieri E, Sigismund S, et al. Unjamming overcomes kinetic and proliferation arrest in terminally differentiated cells and promotes collective motility of carcinoma. Nat Mater. 2019;18(11):1252–63. doi: 10.1038/s41563-019-0425-1 31332337

[3] Ilina O, Gritsenko PG, Syga S, Lippoldt J, La Porta CAM, Chepizhko O, et al. Cell-cell adhesion and 3D matrix confinement determine jamming transitions in breast cancer invasion. Nature Cell Biology. 2020;22(9):1103–15. doi: 10.1038/s41556-020-0552-6 32839548 PMC7502685

[4] Geiger F, Rüdiger D, Zahler S, Engelke H. Fiber stiffness, pore size and adhesion control migratory phenotype of MDA-MB-231 cells in collagen gels. Plos One. 2019;14(11):e0225215. doi: 10.1371/journal.pone.0225215 31721794 PMC6853323

[5] Charras G, Sahai E. Physical influences of the extracellular environment on cell migration. Nature Reviews: Molecular Cell Biology. 2014;15(12):813–24. doi: 10.1038/nrm3897 25355506

[6] Dietrich M, Le Roy H, Bruckner DB, Engelke H, Zantl R, Radler JO, et al. Guiding 3D cell migration in deformed synthetic hydrogel microstructures. Soft Matter. 2018;14(15):2816–26. doi: 10.1039/c8sm00018b 29595213

[7] Harley BA, Kim HD, Zaman MH, Yannas IV, Lauffenburger DA, Gibson LJ. Microarchitecture of three-dimensional scaffolds influences cell migration behavior via junction interactions. Biophysical Journal. 2008;95(8):4013–24. doi: 10.1529/biophysj.107.122598 18621811 PMC2553126

[8] Wu P-H, Gilkes DM, Wirtz D. The Biophysics of 3D Cell Migration. Annual Review of Biophysics. 2018;47(1):549–67.

[9] Caswell PT, Zech T. Actin-Based Cell Protrusion in a 3D Matrix. Trends in Cell Biology. 2018;28(10):823–34. doi: 10.1016/j.tcb.2018.06.003 29970282 PMC6158345

[10] Riching KM, Cox BL, Salick MR, Pehlke C, Riching AS, Ponik SM, et al. 3D collagen alignment limits protrusions to enhance breast cancer cell persistence. Biophysical Journal. 2014;107(11):2546–58. doi: 10.1016/j.bpj.2014.10.035 25468334 PMC4255204

[11] Han W, Chen S, Yuan W, Fan Q, Tian J, Wang X, et al. Oriented collagen fibers direct tumor cell intravasation. Proc Natl Acad Sci U S A. 2016;113(40):11208–13. doi: 10.1073/pnas.1610347113 27663743 PMC5056065

[12] Rubenstein BM, Kaufman LJ. The role of extracellular matrix in glioma invasion: a cellular Potts model approach. Biophysical Journal. 2008;95(12):5661–80. doi: 10.1529/biophysj.108.140624 18835895 PMC2599859

[13] Carey SP, Goldblatt ZE, Martin KE, Romero B, Williams RM, Reinhart-King CA. Local extracellular matrix alignment directs cellular protrusion dynamics and migration through Rac1 and FAK. Integr Biol (Camb). 2016;8(8):821–35. doi: 10.1039/c6ib00030d 27384462 PMC4980151

[14] Ahmadzadeh H, Webster MR, Behera R, Jimenez Valencia AM, Wirtz D, Weeraratna AT, et al. Modeling the two-way feedback between contractility and matrix realignment reveals a nonlinear mode of cancer cell invasion. Proc Natl Acad Sci U S A. 2017;114(9):E1617–e26. doi: 10.1073/pnas.1617037114 28196892 PMC5338523

[15] Provenzano PP, Eliceiri KW, Campbell JM, Inman DR, White JG, Keely PJ. Collagen reorganization at the tumor-stromal interface facilitates local invasion. BMC Med. 2006;4(1):38. doi: 10.1186/1741-7015-4-38 17190588 PMC1781458

[16] Shi Q, Ghosh RP, Engelke H, Rycroft CH, Cassereau L, Sethian JA, et al. Rapid disorganization of mechanically interacting systems of mammary acini. Proc Natl Acad Sci U S A. 2014;111(2):658–63. doi: 10.1073/pnas.1311312110 24379367 PMC3896145

[17] Lee P, Lin R, Moon J, Lee LP. Microfluidic alignment of collagen fibers for in vitro cell culture. Biomedical Microdevices. 2006;8(1):35–41. doi: 10.1007/s10544-006-6380-z 16491329

[18] Ray A, Morford RK, Ghaderi N, Odde DJ, Provenzano PP. Dynamics of 3D carcinoma cell invasion into aligned collagen. Integr Biol (Camb). 2018;10(2):100–12. doi: 10.1039/c7ib00152e 29340409 PMC6004317

[19] Licup AJ, Münster S, Sharma A, Sheinman M, Jawerth LM, Fabry B, et al. Stress controls the mechanics of collagen networks. Proceedings of the National Academy of Sciences. 2015;112(31):9573–8. doi: 10.1073/pnas.1504258112 26195769 PMC4534289

[20] Kopanska KS, Alcheikh Y, Staneva R, Vignjevic D, Betz T. Tensile Forces Originating from Cancer Spheroids Facilitate Tumor Invasion. Plos One. 2016;11(6):e0156442. doi: 10.1371/journal.pone.0156442 27271249 PMC4896628

[21] Park J, Kim DH, Shah SR, Kim HN, Kshitiz, Kim P, et al. Switch-like enhancement of epithelial-mesenchymal transition by YAP through feedback regulation of WT1 and Rho-family GTPases. Nat Commun. 2019;10(1):2797. doi: 10.1038/s41467-019-10729-5 31243273 PMC6594963

[22] Illes B, Fuchs A, Gegenfurtner F, Ploetz E, Zahler S, Vollmar AM, et al. Spatio-selective activation of nuclear translocation of YAP with light directs invasion ofcancer cell spheroids. iScience. 2021;24(3):102185. doi: 10.1016/j.isci.2021.102185 33718837 PMC7921841

[23] Dupont S, Morsut L, Aragona M, Enzo E, Giulitti S, Cordenonsi M, et al. Role of YAP/TAZ in mechanotransduction. Nature. 2011;474(7350):179–83. doi: 10.1038/nature10137 21654799

[24] Thielicke W. The Flapping Flight of Birds: Analysis and Application. Phd thesis, University of Groningen. 2014.

[25] Thielicke W, Stamhuis EJ. PIVlab–Towards User-friendly, Affordable and Accurate Digital Particle Image Velocimetry in MATLAB. Journal of Open Research Software. 2014;2.

[26] Schindelin J, Arganda-Carreras I, Frise E, Kaynig V, Longair M, Pietzsch T, et al. Fiji: an open-source platform for biological-image analysis. Nature Methods. 2012;9(7):676–82. doi: 10.1038/nmeth.2019 22743772 PMC3855844

[27] Schneider CA, Rasband WS, Eliceiri KW. NIH Image to ImageJ: 25 years of image analysis. Nature Methods. 2012;9(7):671–5. doi: 10.1038/nmeth.2089 22930834 PMC5554542

