# Supplementary figures and images for: Directed invasion of cancer cell spheroids inside 3D collagen matrices oriented by microfluidic flow in experiment and simulation
Source: PLoS One. 2022 Mar 1;17(3):e0264571. doi: 10.1371/journal.pone.0264571 (PMC8887745; doi:10.1371/journal.pone.0264571)

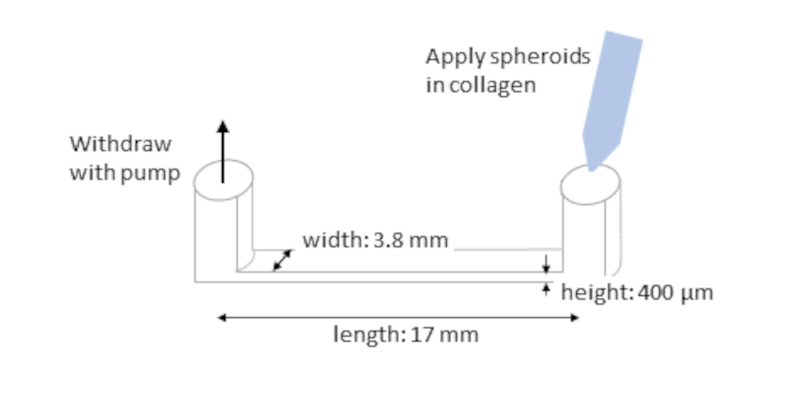

Supplement: S1 Fig — Dimensions as stated; drawing not to scale. (TIF) [file pone.0264571.s001.tif]

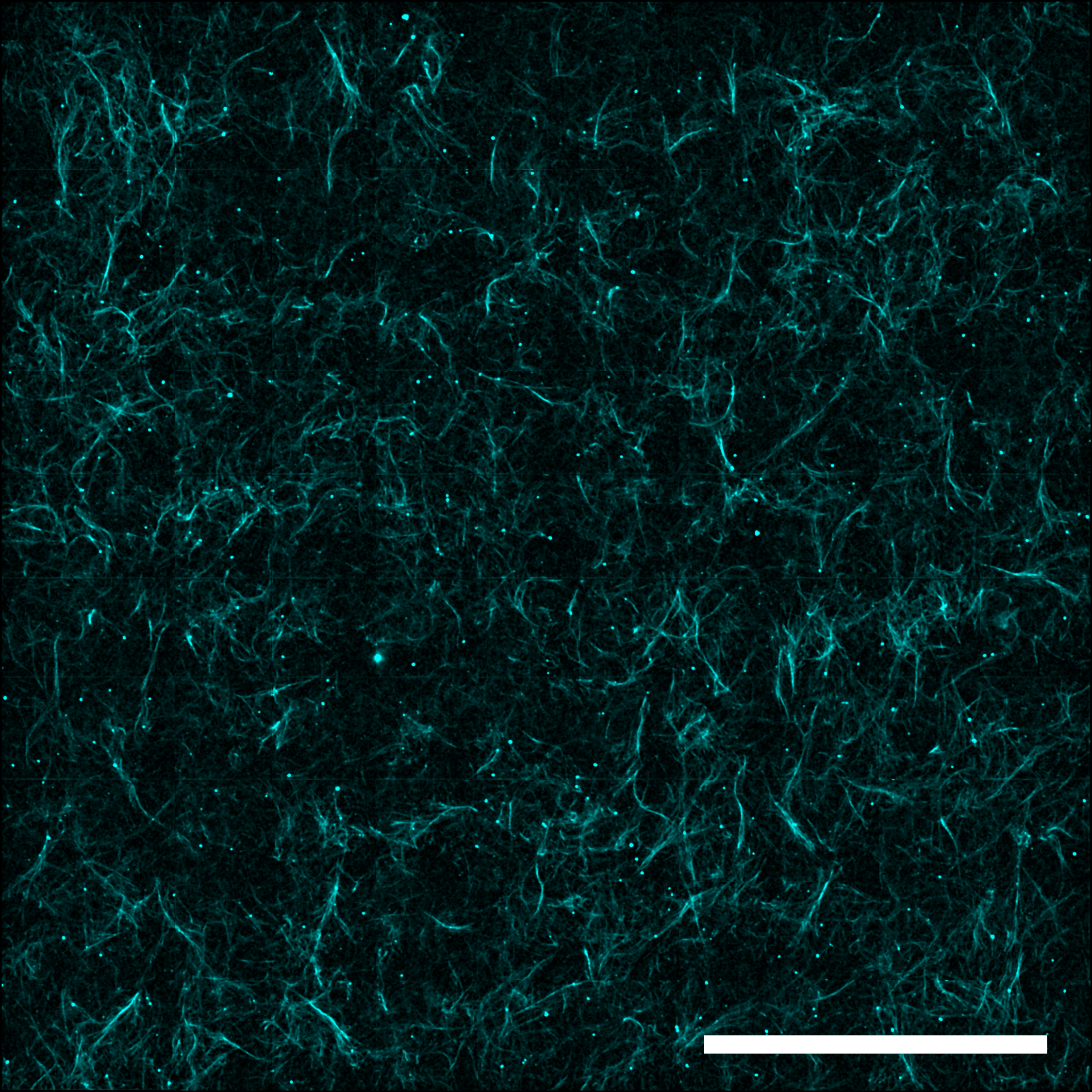

Supplement: S2 Fig — Scale bar = 200 μm. (TIF) [file pone.0264571.s002.tif]

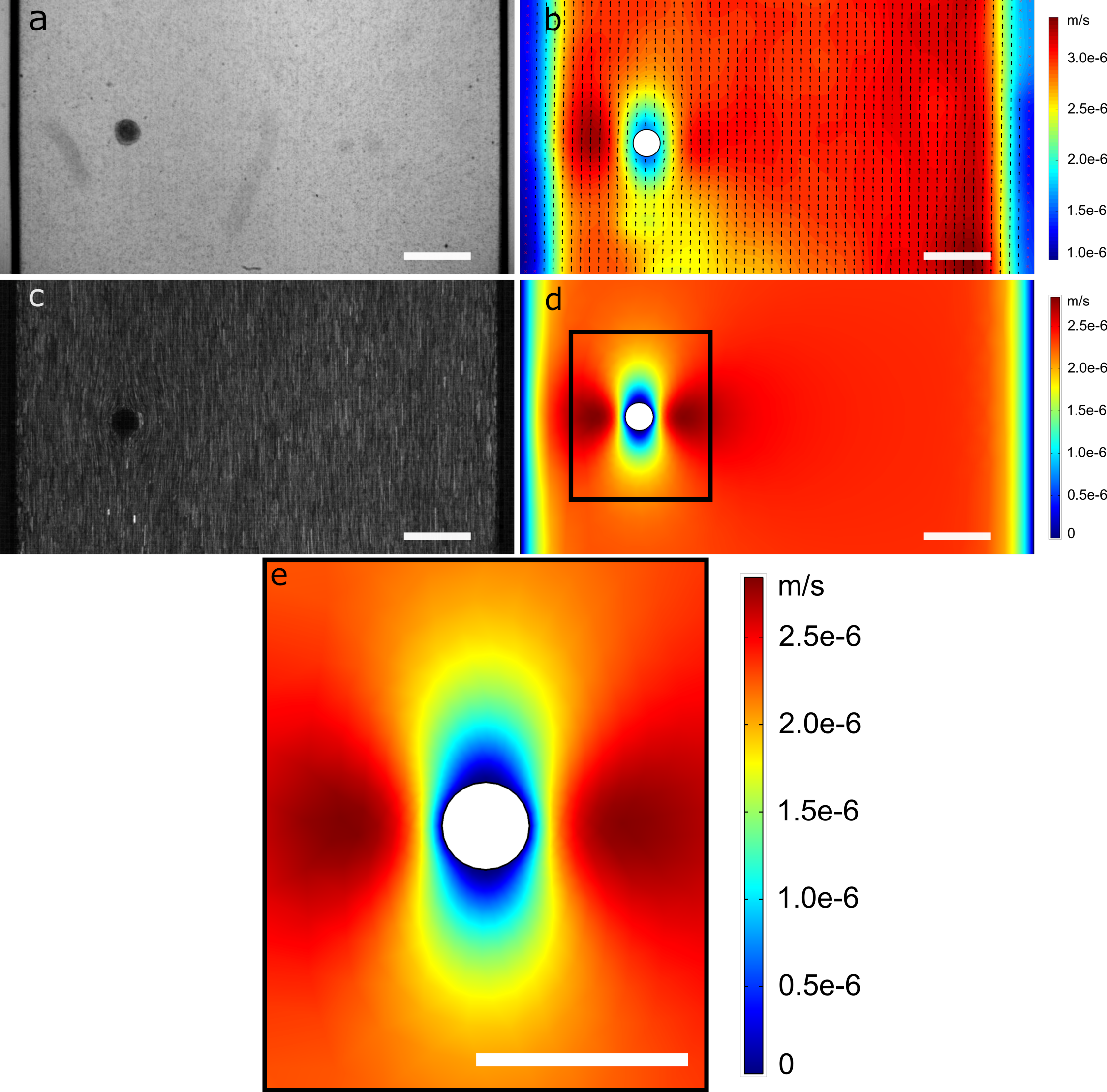

Supplement: S3 Fig — a) Brightfield image of a cell aggregate embedded in a collagen bead mixture without applied flow. b) Calculated velocity magnitude with flow vectors of the beads. c) Flow trajectory of beads around a cell aggregate embedded in collagen during polymerization. d) Simulated velocity field of the collagen mixture around the cell aggregate in the microfluidic channel. e) Zoom in on the immediate surrounding of the cell aggregate in the simulation. Scale bars = 500 μm. (TIF) [file pone.0264571.s003.tif]

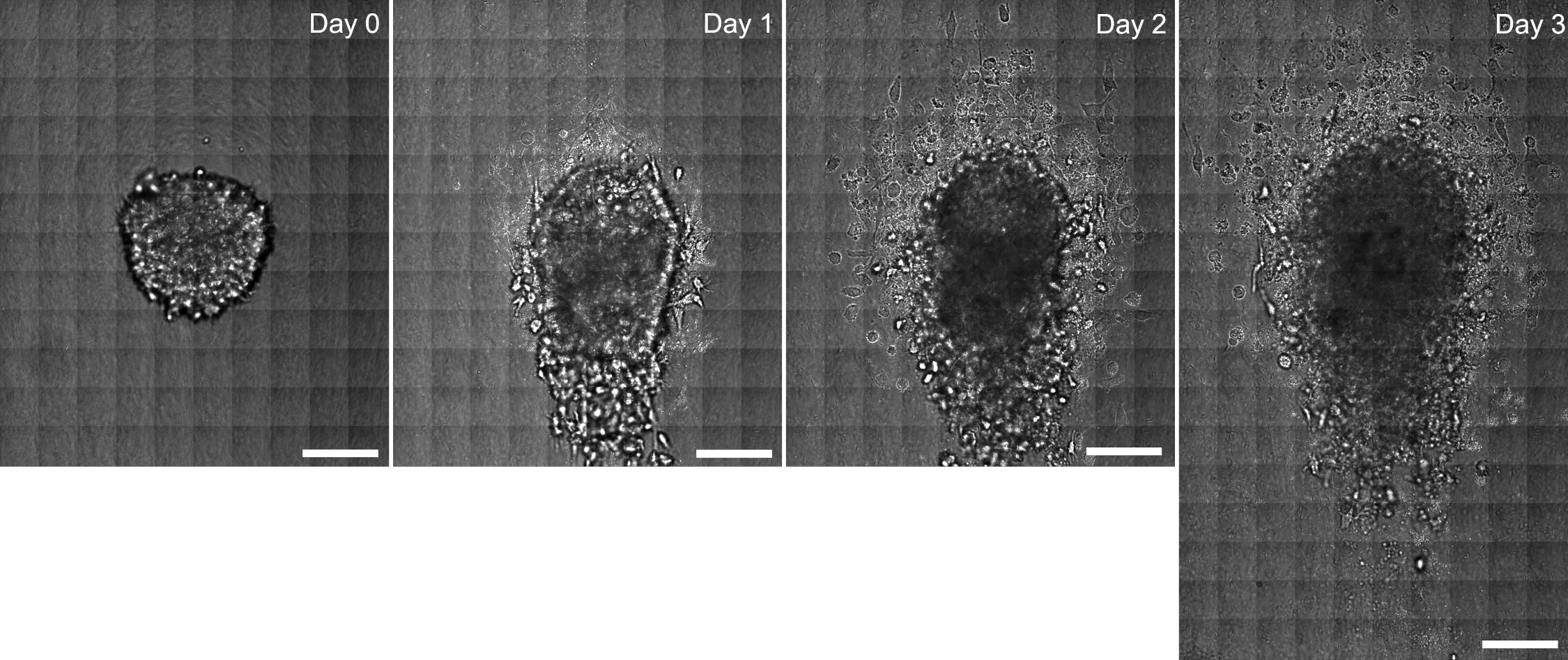

Supplement: S4 Fig — Scale bars = 200 μm. (TIF) [file pone.0264571.s004.tif]

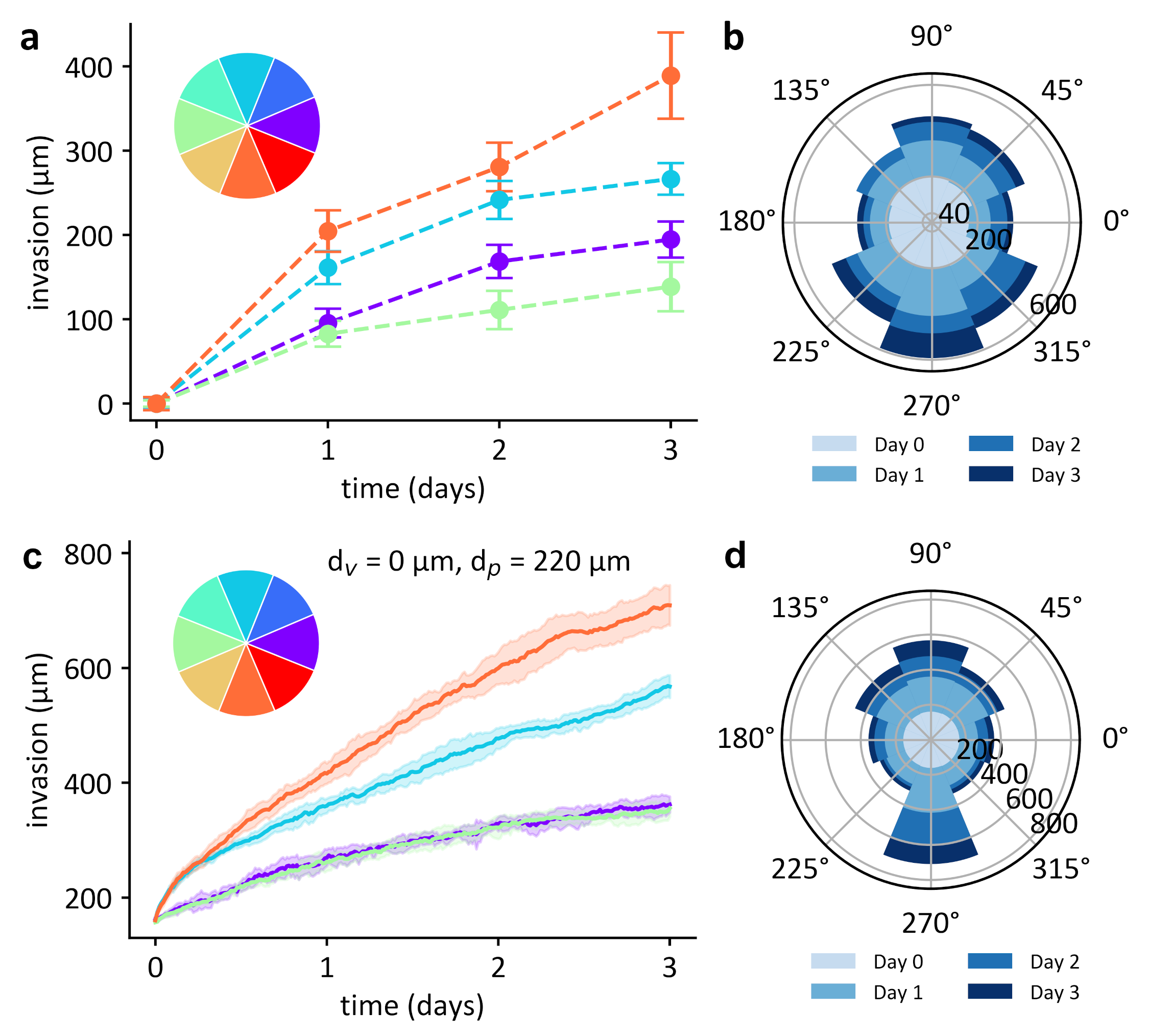

Supplement: S5 Fig — a) and b) Data from experiment: a) Invasion distance of 10 outmost cells in 4 main directions (color-coded directions). The markers are averages of 20 aggregates, errors are standard error of the mean. b) Invasion distance of 10 outmost cells for different directions. The distance travelled per day in each direction is color coded in blue. c) and d) Simulation data (step size parallel to fibers dp = 220 μm, perpendicular to fibers dv = 0 μm): c) Invasion distance of 10 outmost cells in 4 main directions (color-coded directions). The lines are averages of 10 simulations, errors (shades) are standard deviations. d) Invasion distance of 10 outmost cells for different directions. The distance travelled per day in each direction is color coded in blue. (TIF) [file pone.0264571.s005.tif]

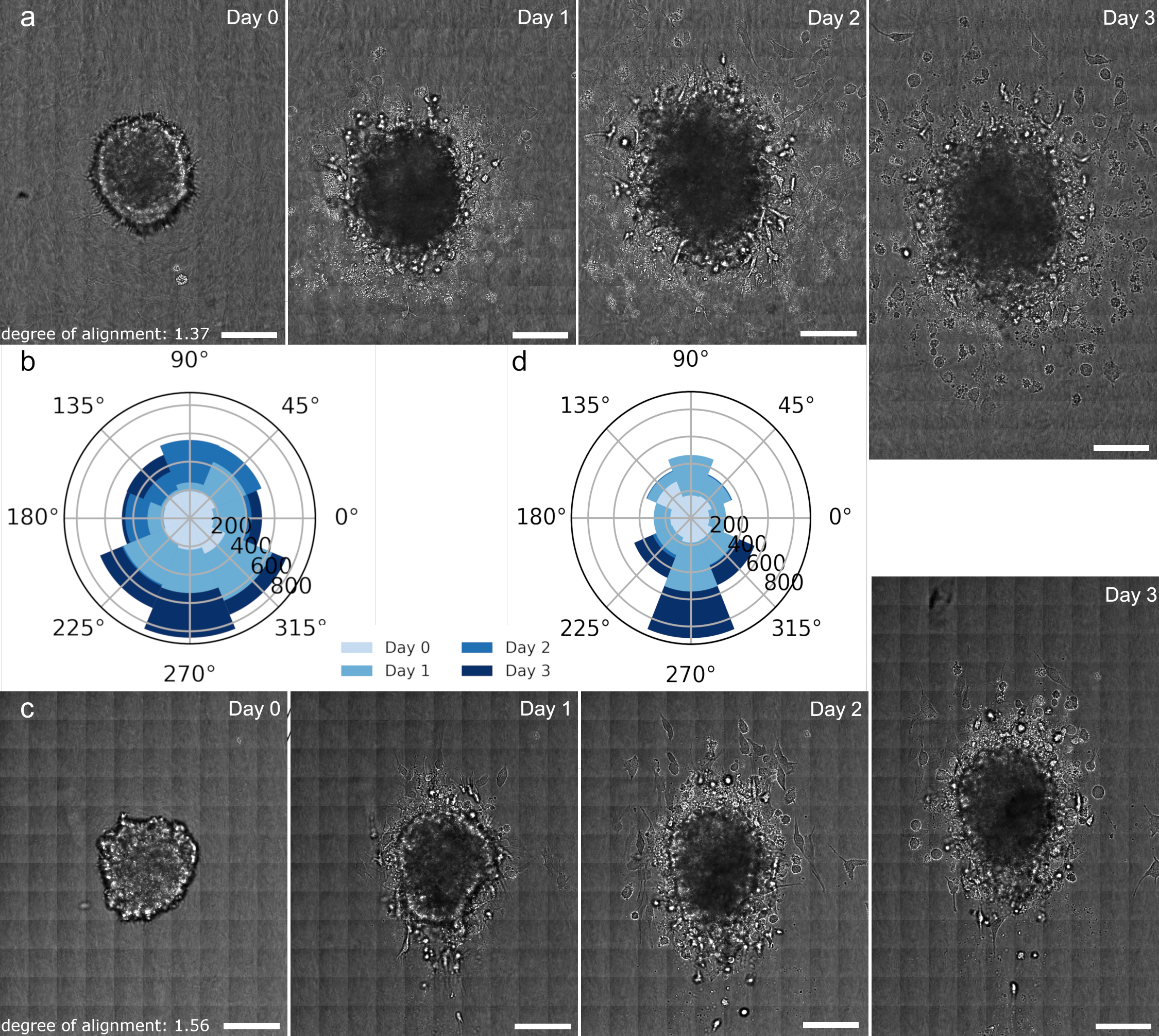

Supplement: S6 Fig — The displayed spheroids provide an overview of experimental variability. a) Spheroid showing a fairly low degree of asymmetry. b) Analysis of directionality of invasion of spheroid displayed in a). c) Spheroid showing fairly strong asymmetry of invasion. d) Analysis of directionality of invasion of spheroid displayed in c). Scale bars = 200 μm. (TIF) [file pone.0264571.s006.tif]

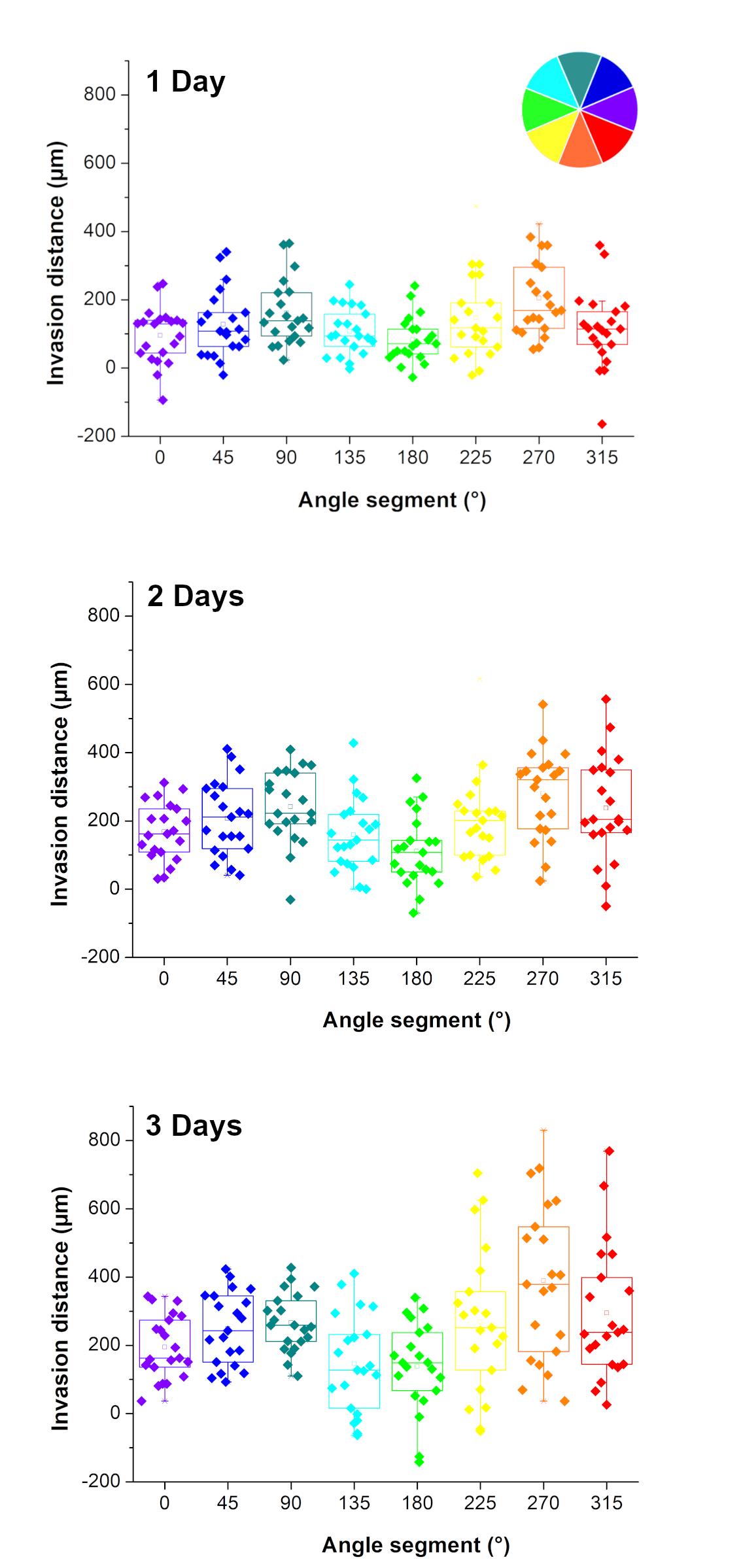

Supplement: S7 Fig — Relative invasion distance travelled by outmost cells within first day, first 2 and 3 days. The respective mean values are represented in Fig 3b. (TIF) [file pone.0264571.s007.tif]

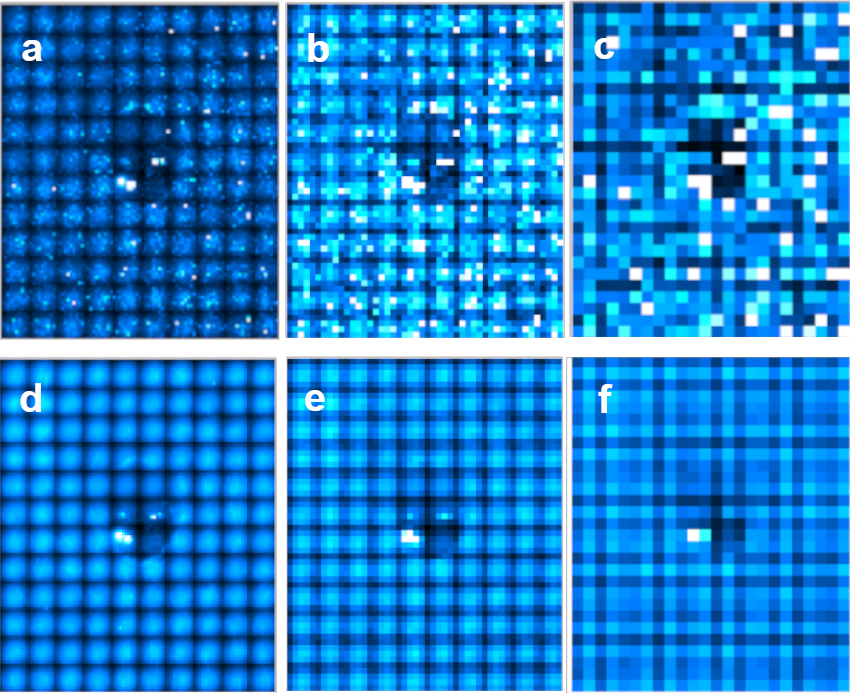

Supplement: S8 Fig — The intensity of the representative fiber image shown in Fig 3 on day 0 at various binnings to assess the density distribution of collagen. a)-c) show maximum intensity binning at various binning factors. d)-f) show mean intensity binning at various binning factors. These exemplary intensity distributions show that the fiber density is homogeneous around the spheroid and not affected by the flow. (TIF) [file pone.0264571.s008.tif]

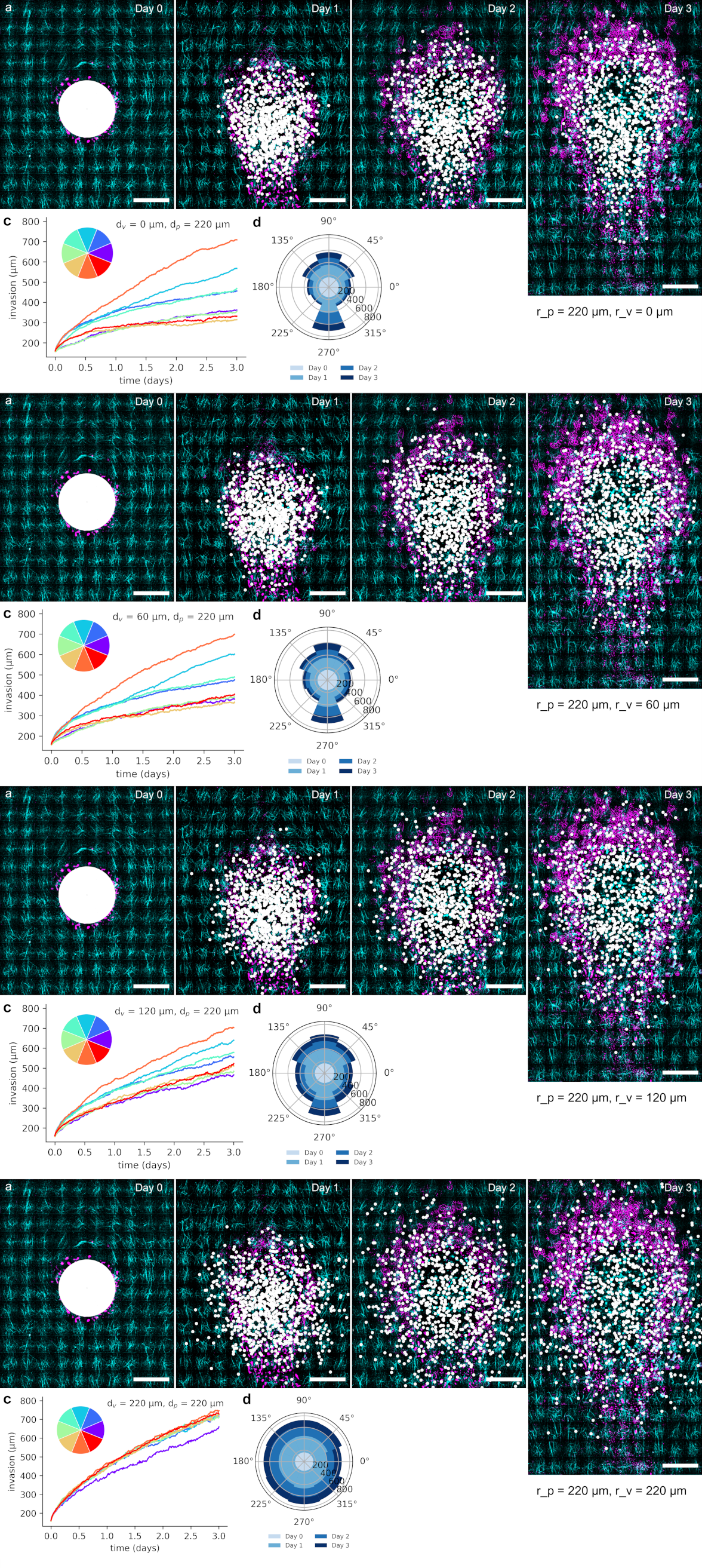

Supplement: S10 Fig — a) Overlayed images of cell invasion in the collagen gel (cyan) during the experiment (magenta) and the simulated migration of cells (white) in a similar environment over the course of 3 days. Parallel step size (r_p) = 220 μm b). Invasion distance of 10 outmost cells in 4 main directions (color-coded directions). d) Invasion distance of 10 outmost cells for different directions. The distance travelled per day in each direction is color coded in blue. Scale bars = 200 μm. (TIF) [file pone.0264571.s010.tif]

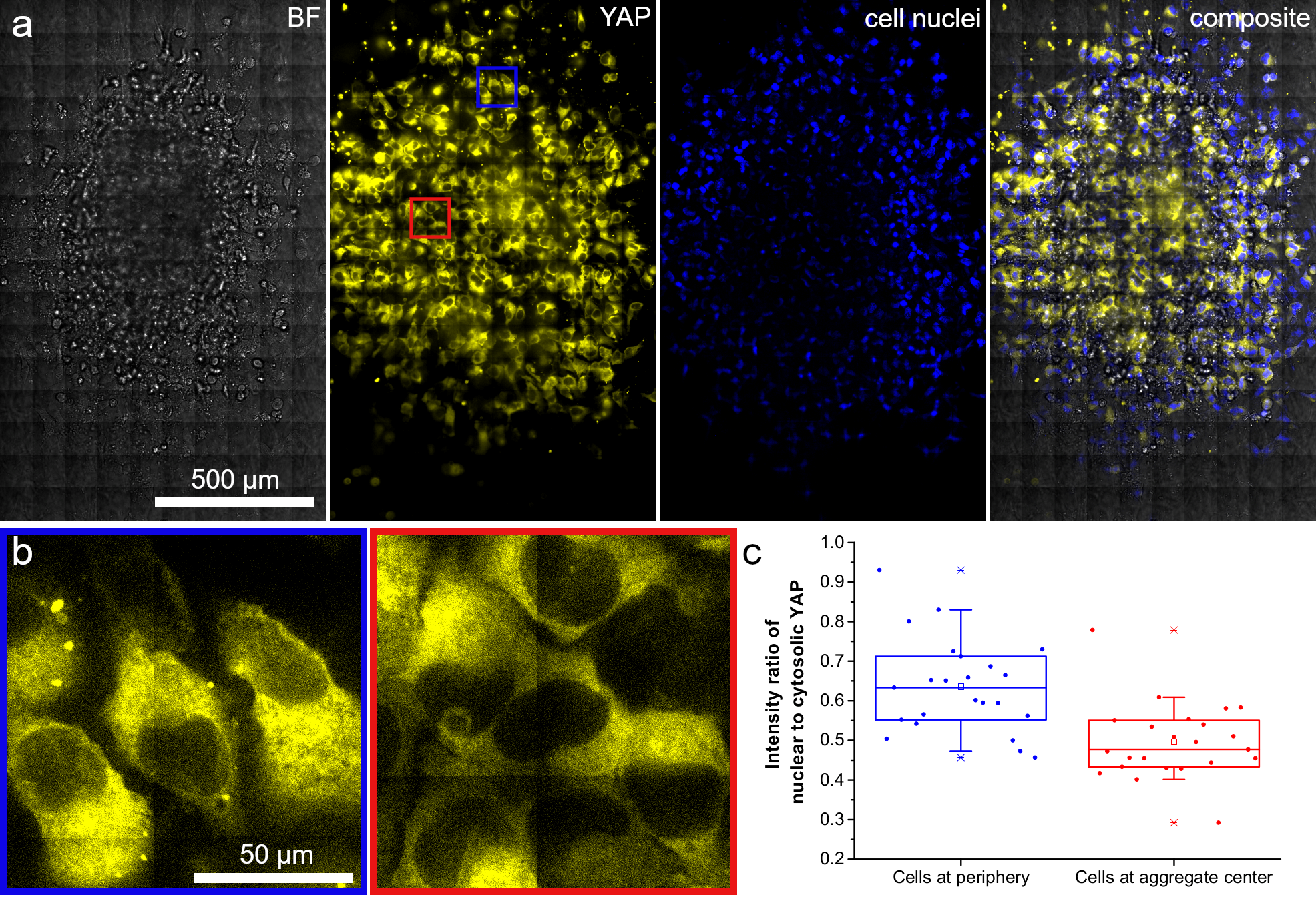

Supplement: S11 Fig — a) Spheroid fixed after 3 days in an aligned collagen gel in a microfluidic channel. The images show brightfield (BF), YAP immuno-staining (YAP, yellow), the nuclei stained with Hoechst 33342 (cell nuclei, blue), and a composite of YAP and Hoechst. b) Zoom-in of YAP stained in cells at the spheroid periphery (blue box) and in the center of the spheroid (red box). c) Ratio of YAP intensity in the cell nuclei and cytosol for cells at the periphery (blue) and cells at the aggregate center (red); p < 10–4. (TIF) [file pone.0264571.s011.tif]
